# Flourishing in chronic disease care: a narrative review and proposed conceptual framework

**DOI:** 10.3389/fpsyg.2026.1915817

**Published:** 2026-08-20

**Authors:** Zhenzhen Feng, Qin Yang

**Affiliations:** 1 Sichuan Provincial People’s Hospital, University of Electronic Science and Technology of China, Chengdu, China; 2 School of Medicine, University of Electronic Science and Technology of China, Chengdu, China

**Keywords:** adaptation, chronic diseases, conceptual framework, flourishing, positive mental health

## Abstract

The growing burden of chronic diseases has increased interest in both psychological distress and positive psychological functioning among people living with chronic conditions. In this context, flourishing may be understood as the capacity to maintain positive functioning, meaning, social connectedness, and personal growth while adapting to long-term illness. This narrative review examines the conceptualization, theoretical foundations, current evidence, measurement instruments, and factors associated with flourishing in chronic disease populations. Insights from Keyes’ Mental Health Continuum, the complete state model of mental health, the PERMA model, and evidence on contextual and psychological factors are integrated to propose a model-informed conceptual framework for future research and intervention development. The proposed framework emphasizes the complementary goals of reducing psychological distress and strengthening positive psychological resources, thereby broadening chronic disease care from symptom control toward patient-centred approaches that also support positive functioning, adaptation, and self-management. Further empirical research is required to evaluate the conceptual validity, feasibility, and applicability of this framework across chronic disease populations.

## 1 Introduction

The global burden of chronic diseases continues to pose substantial challenges to health systems, long-term care, and quality of life worldwide. In 2021, noncommunicable diseases, including cardiovascular diseases, cancers, chronic respiratory diseases, and diabetes, were responsible for approximately 43.8 million deaths globally (Li et al., 2025). Meanwhile, the number of people aged 60 years or older is projected to increase from 1.0 billion in 2020 to 1.4 billion by 2030 (Noto, 2023). This demographic transition is likely to further increase demands for chronic disease management, healthcare delivery, and long-term care.

Chronic diseases often involve persistent symptoms, functional limitations, and ongoing self-management demands. People living with these conditions are therefore at increased risk of psychological distress, particularly anxiety and depression (Baxter and Sirois, 2025; Scott et al., 2023). Such distress may further complicate treatment adherence, self-management, and adaptation to long-term illness (Scott et al., 2023).

Despite these challenges, people living with chronic diseases may still experience positive psychological functioning and adapt meaningfully to illness (Schmidt-Sane et al., 2023; Allaire et al., 2022). Flourishing provides a complementary perspective for understanding these positive aspects of living with chronic disease by focusing on the presence of positive emotional, psychological, and social functioning rather than solely on psychological distress (Keyes, 2002; Huppert and So, 2013).

However, evidence on flourishing in chronic disease populations remains dispersed across different disease groups and research contexts. A focused synthesis is therefore needed to clarify how flourishing has been conceptualized and assessed in these populations, integrate the available disease-specific evidence and associated factors, and inform the development of a conceptual framework for future research and intervention. This narrative review therefore aims to: (1) clarify the conceptual and theoretical foundations of flourishing; (2) summarize current evidence and measurement approaches in chronic disease populations; (3) synthesize factors associated with flourishing; and (4) propose a model-informed conceptual framework to guide future research and intervention development.

## 2 Methods

### 2.1 Review design

This study was conducted as a narrative review with an integrative orientation. This approach was selected to bring together conceptual, theoretical, measurement, and empirical literature on flourishing across different chronic disease populations and to support the development of a model-informed conceptual framework. The conduct and reporting of the review were informed by Sukhera’s guidance on rigorous and transparent narrative reviews (Sukhera, 2022).

### 2.2 Literature identification and selection

Relevant literature was identified through an iterative search of PubMed, Web of Science, Embase, CINAHL, the China National Knowledge Infrastructure, and Wan Fang Data. Searches covered the period from database inception to December 2025, using “flourishing” and its Chinese equivalent as the core search terms. Database searches were supplemented by backward screening of the reference lists of relevant publications and forward citation tracking of key papers. English- and Chinese-language publications were considered.

For the empirical literature concerning chronic disease populations, studies were considered when they involved adults living with chronic physical conditions and explicitly assessed flourishing. Studies involving only children or adolescents were not considered. The chronic disease populations represented in the identified literature were determined by the available studies rather than being specified in advance.

The literature was narratively organized and synthesized according to the conceptualization and theoretical foundations of flourishing, measurement instruments, evidence across chronic disease populations, associated contextual and psychological factors, and their relevance to the proposed conceptual framework. The review was intended to provide an integrative and conceptually informative synthesis rather than an exhaustive mapping of all eligible publications. The literature identification, selection, and synthesis process is summarized in Table 1.

**Table 1:** Summary of the literature identification and synthesis process.

Methodological component	Description
Databases	PubMed, Web of Science, Embase, CINAHL, CNKI, Wan Fang Data
Search period	From database inception to December 2025
Core search term	“Flourishing” and its Chinese equivalent
Supplementary identification	Backward reference-list screening and forward citation tracking of key publications
Language	English and Chinese
Empirical literature focus	Studies involving adults with chronic physical conditions in whom flourishing was explicitly assessed
Exclusion relevant to population	Studies involving only children or adolescents
Types of literature considered	Conceptual, theoretical, measurement, review, and empirical publications relevant to flourishing and chronic disease care.
Synthesis approach	Narrative synthesis organized around conceptual foundations, theoretical models, measurement instruments, evidence across chronic disease populations, associated factors, and framework development.

## 3 Conceptualizing flourishing in chronic disease

Flourishing is commonly conceptualized as a high or optimal state of well-being and positive mental health (Keyes, 2002; Huppert and So, 2013). Rooted in the Aristotelian concept of eudaimonia (Waterman, 1993), it refers not merely to the absence of mental illness or distress, but to the presence of positive feelings, meaning, and effective psychological and social functioning (Keyes, 2002; Huppert and So, 2013). In the context of chronic disease, flourishing may be understood as the capacity to maintain positive functioning, meaning, adaptation, and personal growth while living with long-term illness and its associated challenges (Schmidt-Sane et al., 2023; Allaire et al., 2022).

No universally accepted definition of flourishing has been established. Existing conceptualizations nevertheless converge on the view that flourishing is multidimensional and involves high levels of emotional, psychological, and social well-being, together with meaningful functioning and personal development (Rule et al., 2024). In this review, flourishing is used as an umbrella term for multidimensional positive functioning. This usage recognizes that the Flourishing Scale, the Mental Health Continuum-Short Form, the PERMA-Profiler, and the Flourish Index/Secure Flourish Index arise from partly different conceptual traditions and should not be treated as interchangeable measures.

## 4 Theoretical foundations of flourishing

### 4.1 Keyes’ mental health continuum and complete state model

Keyes’ Mental Health Continuum conceptualizes positive mental health as a continuum ranging from languishing to flourishing (Keyes, 2002). Within this continuum, flourishing reflects high levels of emotional well-being together with optimal psychological and social functioning, whereas languishing reflects low levels of positive feelings and functioning.

Keyes’ later complete state model further distinguishes positive mental health from mental illness as two related but distinct continua (Keyes, 2005). Within this model, flourishing denotes a high level of positive mental health, whereas complete mental health refers to the combination of flourishing and a low level of mental illness. This distinction is particularly relevant to chronic disease care because alleviating anxiety, depression, or other psychological symptoms does not necessarily result in high positive mental health. Conversely, the presence of mental illness or psychological distress does not necessarily preclude all aspects of well-being.

### 4.2 The PERMA model

The PERMA model was introduced by Seligman as part of his theory of well-being, which expanded his earlier authentic happiness theory into a broader multidimensional account of flourishing (Seligman, 2011). The model identifies five measurable and potentially cultivable domains of well-being: positive emotion, engagement, relationships, meaning, and accomplishment (Seligman, 2011). Together, these domains provide a practical structure for describing positive functioning and identifying potential areas through which flourishing may be strengthened.

### 4.3 Complementarity and conceptual boundaries

Keyes’ framework and the PERMA model offer complementary, but not identical, perspectives on flourishing. Keyes’ work explains why psychological care should address both mental illness and positive mental health, whereas the PERMA model identifies specific domains of positive functioning that may potentially be strengthened. Their integration therefore supports a dual emphasis on reducing psychological distress and cultivating positive psychological functioning.

However, neither model alone fully captures the disease-specific stressors, functional limitations, treatment demands, and contextual resources that shape the experiences of people living with chronic diseases. Keyes’ framework provides an overarching account of mental health status but offers limited guidance regarding specific pathways through which flourishing may be promoted. In contrast, the PERMA model provides potentially modifiable domains of well-being but gives less explicit attention to the clinical and contextual conditions associated with chronic illness. These conceptual boundaries support the need to integrate the two theoretical perspectives with empirical evidence on the contextual and psychological factors associated with flourishing.

## 5 Current evidence on flourishing among patients with chronic diseases

In recent years, flourishing has received increasing attention in healthcare research as an indicator of positive mental health among people living with chronic diseases. Existing evidence suggests that the level of flourishing varies considerably across disease populations. In adults with immune-mediated inflammatory diseases, including inflammatory bowel disease, multiple sclerosis, and rheumatoid arthritis, more than half of participants were classified as having flourishing mental health, with broadly comparable levels across the three disease groups (Almweisheer et al., 2023). Among head and neck cancer survivors, lower flourishing was associated with greater psychological distress, symptom burden, functional impairment, and poorer quality of life (Harris et al., 2022).

Other studies have reported varying flourishing scores across specific chronic disease populations. Among 362 postoperative patients with thyroid cancer, the mean item score on the Flourishing Scale was 4.89 ± 0.43, which the original authors interpreted as indicating room for further improvement in flourishing (Huili et al., 2025). In 332 patients with diabetic retinopathy, most participants reported low or moderate flourishing, and a greater number of comorbid chronic diseases was associated with lower flourishing (Gang et al., 2025). Similarly, a multicenter study of 376 patients undergoing maintenance hemodialysis reported a mean PERMA-Profiler flourishing score of 6.28 ± 1.76, indicating a moderate level of flourishing (Zeng X. Q. et al., 2025).

These findings support the conclusion that flourishing in chronic disease populations is heterogeneous rather than uniformly low. Differences may reflect disease type, symptom and treatment burden, multimorbidity, functional status, measurement approach, and sociocultural context. Direct comparisons should nevertheless be interpreted cautiously because the MHC-SF, Flourishing Scale, and PERMA-Profiler assess partly different constructs and use different scoring and classification methods. Most reported associations are observational and should not be interpreted as causal.

## 6 Measurement instruments

Several instruments have been used to assess flourishing or closely related forms of positive mental health in chronic disease populations. Table 2 summarizes their principal characteristics and applications.

**Table 2:** Measurement instruments for assessing flourishing among patients with chronic diseases.

Instru-ment	Developer	Chronic disease populations	Conceptual framework	Scoring	Interpretation
FS	Diener et al. (2010)	Diabetic retinopathy (Gang et al., 2025); postoperative thyroid cancer (Huili et al., 2025); spinal cord injury (Allaire et al., 2022);breast cancer (Cerezo et al., 2024)	Eight-item global measure covering purpose and meaning, supportive relationships, engagement, contribution to others, competence, self-respect, optimism, and social respect	Seven-point response scale	Higher mean or total scores indicate greater flourishing. Cut-offs used in individual studies are not universally established.
MHC-SF	Keyes (2002)	Immune-mediated diseases (Almweisheer et al., 2023); arthritis (Fuller-Thomson et al., 2023)	Emotional, psychological, and social well-being (14 items)	Six-point frequency scale	Can be scored continuously; categorical classification as flourishing, moderate mental health, or languishing follows the established symptom-frequency algorithm.
PERMA-Profiler	Butler and Kern (2016)	Maintenance hemodialysis (Zeng X. Q. et al., 2025); postoperative thyroid cancer (Chonglong et al., 2025)	Includes the five PERMA domains, together with overall well-being, negative emotion, loneliness, and physical health; 9 domains in total	Items scored from 0 to 10	Domain and overall profile scores are calculated according to the instrument guidance; higher scores indicate stronger functioning in the relevant domains.
FI/SFI	VanderWeele (2017)	Head and neck cancer survivors (Harris et al., 2022)	FI: happiness and life satisfaction, mental and physical health, meaning and purpose, character and virtue, and close social relationships; SFI additionally includes financial and material stability	Items scored from 0 to 10	Higher scores indicate more favorable functioning across the assessed life domains.

FS, Flourishing Scale; MHC-SF, Mental Health Continuum–Short Form; PERMA, Positive Emotion, Engagement, Relationships, Meaning, and Accomplishment; FI, Flourishing Index; SFI, Secure Flourishing Index.

### 6.1 The flourishing scale

The Flourishing Scale (FS) consists of eight items and has a unidimensional structure, with all items designed to assess flourishing as an overall construct. Owing to its brevity and efficiency, the FS is suitable for rapid assessment and can effectively capture individuals’ general level of flourishing. However, because of its unidimensional nature, the FS provides limited information on the specific psychological components underlying flourishing.

### 6.2 The mental health continuum-short form

The MHC-SF is a 14-item measure of emotional, psychological, and social well-being (Keyes, 2002). It can provide continuous domain scores and categorical classifications of flourishing, moderate mental health, and languishing. Because the categorical algorithm is based on the frequency of well-being symptoms rather than a simple total-score cut-off, studies should report the scoring approach clearly.

### 6.3 The PERMA-profiler

The PERMA-Profiler was developed by Butler and Kern based on Seligman’s PERMA model. It measures positive emotion, engagement, relationships, meaning, and accomplishment, and includes supplementary items assessing overall well-being, negative emotion, loneliness, and physical health (Butler and Kern, 2016). These supplementary items broaden the assessment beyond the five core PERMA domains and allow examination of related aspects of well-being and distress. Its multidimensional structure enables a more detailed assessment of individuals’ psychological characteristics. Because the applicability of the PERMA-Profiler may vary across patient populations, further validation is needed when it is applied to specific chronic disease groups.

### 6.4 The flourish index and secure flourish index

The 10-item Flourish Index (FI) and the 12-item Secure Flourish Index (SFI), proposed by VanderWeele in 2017, assess flourishing using a multidomain framework (VanderWeele, 2017). These instruments cover multiple domains of human flourishing, including happiness and life satisfaction, mental and physical health, meaning and purpose, character and virtue, and close social relationships, with the SFI additionally including financial and material stability. Compared with the FS, MHC-SF, and PERMA-Profiler, the FI and SFI adopt a broader whole-person framework and may be particularly useful when flourishing is conceptualized beyond psychological well-being alone.

Overall, existing instruments for measuring flourishing differ in item content, dimensional structure, and theoretical focus, and no unified measurement standard has been established. This heterogeneity may be explained by several factors. At the theoretical level, different conceptual frameworks of flourishing have not been fully integrated, resulting in conceptual variability across instruments. At the methodological level, the applicability of existing instruments may vary across patient populations; therefore, their dimensional structures, reliability, and validity should be further examined in diverse chronic disease populations. At the disease level, chronic diseases vary substantially in symptom burden, treatment demands, functional limitations, and disease trajectories; therefore, a generic flourishing instrument may not fully capture disease-specific characteristics among patients with different chronic conditions.

## 7 Associated factors of flourishing among patients with chronic diseases

Flourishing in chronic disease populations is associated with factors operating at multiple levels. For conceptual clarity, the evidence can be grouped into contextual and disease-related factors, and psychological processes and individual resources. These categories overlap and should not be interpreted as independent causal layers.

### 7.1 External factors

External factors mainly include age, marital status, economic status, social support, and disease-related characteristics. These factors do not necessarily determine flourishing directly, but they may influence patients’ access to resources, ability to cope with stress, and capacity to maintain a sense of meaning in life, thereby indirectly affecting positive mental health.

Age has shown inconsistent associations with flourishing across disease groups. Some adults aged 50 years or older with cancer maintained complete mental health despite their diagnosis (Fuller-Thomson and West, 2019), whereas younger adults undergoing maintenance hemodialysis reported higher flourishing than older adults in another study (Tianyu et al., 2024). This discrepancy may be related to differences in disease type, treatment burden, and the degree of functional limitation. However, among older patients receiving long-term dialysis, continuous treatment, impaired physical functioning, and frailty may impose additional challenges to psychological adaptation (Yin et al., 2025).

Marital status and family support are also important social factors associated with flourishing. Studies have shown that married patients are more likely to achieve flourishing than those who are divorced, widowed, or single (Gang et al., 2025; Fuller-Thomson and West, 2019). In the context of chronic disease, partners may help patients reduce stress and enhance positive emotions through dyadic coping strategies, such as emotional support, shared decision-making, and collaborative problem-solving (Hou et al., 2025). In addition, intimate relationships and flourishing may have a reciprocal association: high-quality couple interactions may promote flourishing, while higher levels of flourishing may in turn improve relationship quality (Sanri et al., 2021).

Economic status is also associated with flourishing. Lower income has been observed to be associated with lower levels of flourishing among patients with thyroid cancer, maintenance hemodialysis, and head and neck cancer (Harris et al., 2022; Huili et al., 2025; Tianyu et al., 2024). Chronic disease treatment and rehabilitation often involve long-term medical expenses and care-related costs. Financial stress may not only increase negative emotions such as anxiety and depression, but also limit patients’ access to medical and rehabilitation resources, thereby weakening their sense of meaning, accomplishment, and control over life.

Social support from family members, peers, friends, and healthcare professionals is one of the most consistently identified contextual resources. Support may provide emotional reassurance, practical assistance, information, and a sense of belonging. Associations between social support and flourishing have been reported in spinal cord injury, inflammatory bowel disease, breast cancer, and maintenance hemodialysis populations (Allaire et al., 2022; Tianyu et al., 2024; Ran et al., 2025; Krömeke and Shani, 2025). Because most evidence is observational, the direction and mechanisms of these relationships remain uncertain.

Disease-related burden is also relevant. Multimorbidity, long-term treatment, symptom burden, and functional limitations may place sustained demands on psychological resources. A greater number of comorbid chronic diseases was associated with lower flourishing in diabetic retinopathy (Gang et al., 2025). Among postoperative patients with thyroid cancer, those receiving ^131^I adjuvant therapy reported lower levels of flourishing than those who did not receive this treatment (Huili et al., 2025). Among patients undergoing maintenance hemodialysis, a longer dialysis duration was associated with lower flourishing levels (Zeng X. Q. et al., 2025). These associations identify potentially vulnerable groups but do not establish that treatment exposure or disease duration directly causes lower flourishing.

### 7.2 Psychological mechanisms

Compared with external factors, psychological mechanisms more directly reflect patients’ cognitive, emotional, and behavioral responses to illness and life events. Existing evidence suggests that negative psychological states, positive psychological traits, emotion regulation, personality characteristics, illness identity, and early-life experiences may all be associated with flourishing among patients with chronic diseases.

Anxiety, depression, and other forms of psychological distress are generally associated with lower flourishing. Among head and neck cancer survivors, lower flourishing was associated with more severe anxiety and depressive symptoms (Harris et al., 2022). This evidence reinforces the distinction between symptom reduction and flourishing: distress is relevant to flourishing, but the absence of distress alone does not constitute positive mental health.

Hope, resilience, self-compassion, psychological flexibility, and adaptive coping have been identified as potentially protective resources (Schmidt-Sane et al., 2023; Huili et al., 2025; Gang et al., 2025). These factors may support adaptation, meaning-making, and persistence in self-management. In a qualitative study of chronic illness in South Africa, self-compassion and supportive social contexts featured prominently in accounts of living well despite illness (Schmidt-Sane et al., 2023).

Emotion regulation may also be relevant to flourishing because it shapes how individuals manage positive and negative emotional experiences during chronic illness. In patients receiving maintenance hemodialysis, regulatory emotional self-efficacy has been reported to be associated with flourishing and perceived social support, suggesting that emotional self-regulation may represent a potential psychological resource in this population (Zeng X. et al., 2025).

Personality characteristics may also influence how patients interpret and respond to illness-related events. Gang et al. (2025) found that higher levels of conscientiousness, agreeableness, and openness were associated with greater flourishing among patients with diabetic retinopathy. These personality characteristics may enable patients to demonstrate better self-regulation, psychological flexibility, and help-seeking tendencies when facing disease-related stress, thereby making it easier for them to maintain positive emotions and adaptive behaviors. Therefore, personality traits may provide useful information for identifying different psychological support needs, although clinical application should still be based on a comprehensive assessment of the patient’s disease context and psychological status.

Illness identity describes how a chronic condition is incorporated into an individual’s sense of self. In patients with inflammatory bowel disease, specific illness-identity dimensions have been associated with flourishing and health-related quality of life (Krömeke and Shani, 2025). These findings suggest that the way individuals integrate illness into their self-concept may be relevant to positive mental health, although the direction and role of different illness-identity dimensions require careful interpretation.

Early-life adverse experiences may have a long-term impact on flourishing in adulthood. Fuller-Thomson et al (Fuller-Thomson and West, 2019) found that cancer patients with a history of childhood physical abuse were less likely to achieve flourishing in adulthood, and this association remained significant after adjustment for multiple confounding factors. Although research on the relationship between early-life experiences and flourishing among patients with chronic diseases remains relatively limited, adverse childhood experiences are closely related to adult mental health. Future studies may therefore further examine the long-term effects of early psychological trauma on positive mental health among patients living with chronic diseases.

Overall, flourishing among patients with chronic diseases is not determined by a single factor, but rather by the combined effects of external resources, disease burden, and internal psychological mechanisms. External factors, such as social support, economic status, and disease burden, provide the resource or stress context for patients, whereas internal psychological factors, such as resilience, self-compassion, hope, emotion regulation, and illness identity, influence how patients interpret, cope with, and integrate their illness experience. In other words, external resources often need to be transformed through internal psychological mechanisms before they can ultimately affect flourishing.

## 8 Proposed model-informed conceptual framework

### 8.1 Rationale and construction

From the perspective of chronic disease care, Keyes’ Mental Health Continuum and complete state model clarify the need to address psychological distress and positive mental health as related but distinct dimensions. The PERMA model identifies domains of positive functioning that may be strengthened, but it does not explicitly incorporate many disease-related and contextual constraints. The proposed framework therefore integrates these theoretical perspectives with the associated factors summarized in this review. The framework is intended to organize hypotheses and guide future research; it is not a validated causal model or a tested intervention protocol.

### 8.2 Components and proposed pathways

The framework includes four interconnected components. First, contextual and disease-related factors include social support, economic resources, symptom and treatment burden, multimorbidity, and functional limitations. Second, psychological processes and potentially modifiable resources include emotion regulation, resilience, self-compassion, hope, coping, illness identity, meaning-making, and social connectedness. Third, two complementary intervention pathways are proposed: a distress-reduction pathway addressing anxiety, depression, stress, and related psychological symptoms, and a positive-functioning pathway strengthening PERMA-oriented domains and other positive psychological resources. Fourth, hypothesized outcomes include higher flourishing, better quality of life, and improved self-management or treatment adherence.

**Figure 1:**
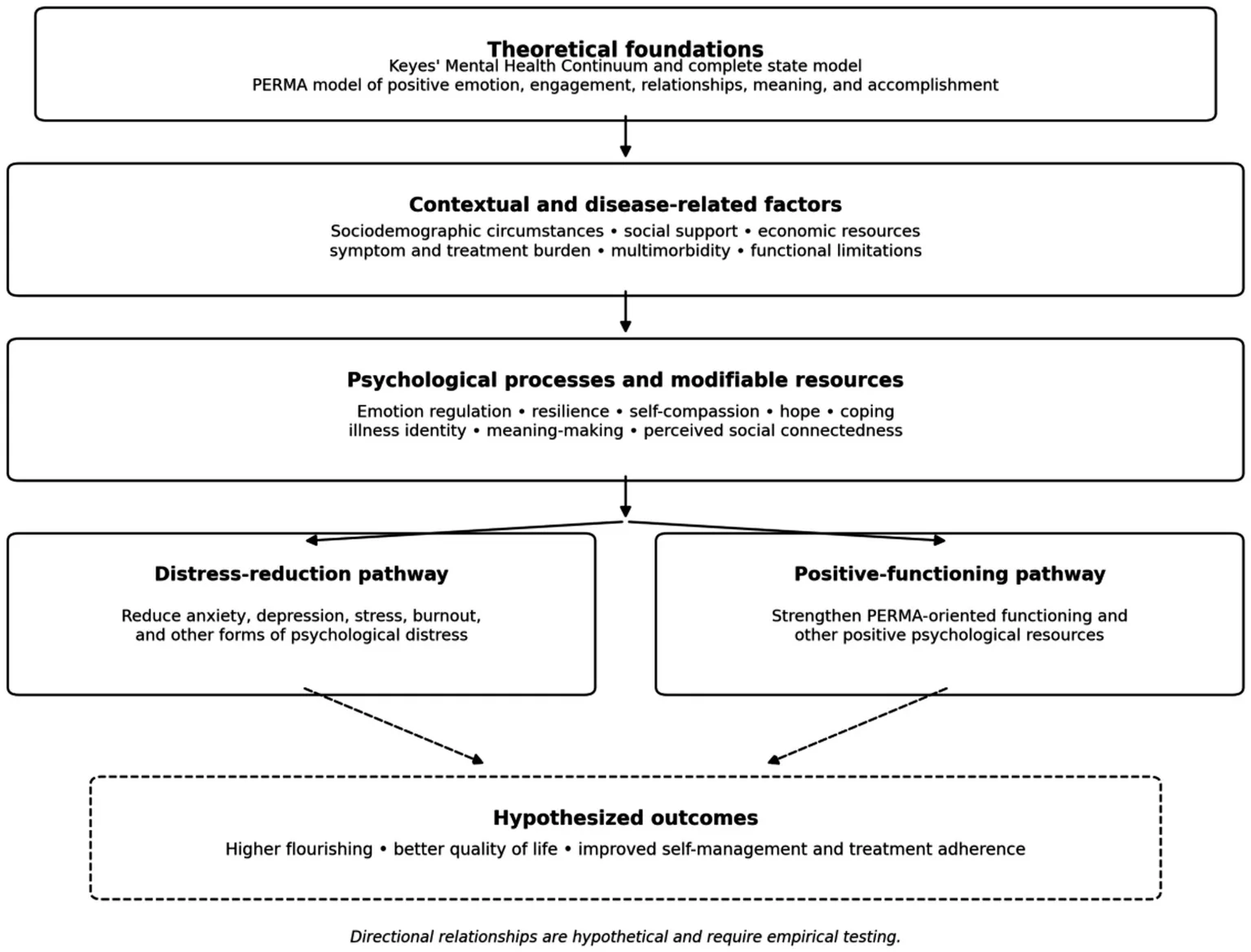
Proposed model-informed conceptual framework for flourishing among people living with chronic diseases. Solid and dashed arrows represent proposed, not established, relationships. The framework requires conceptual validation, stakeholder input, and empirical testing before clinical application.

The order and direction of these relationships are provisional. Contextual factors may influence psychological processes, but reciprocal and feedback relationships are also plausible. Similarly, improvement in flourishing may affect social relationships, coping, and self-management rather than functioning solely as an endpoint. Prospective and experimental studies are required to determine which pathways are causal, modifiable, and clinically meaningful (see Figure 1).

### 8.3 Framework validation priorities

Initial validation should include expert review and involvement of people living with chronic diseases, caregivers, and healthcare professionals to assess relevance, clarity, acceptability, and missing domains. Subsequent studies should evaluate content validity, feasibility, temporal relationships, potential mediators and moderators, and the responsiveness of candidate outcome measures. Only after such work should the framework be translated into a structured intervention and evaluated in feasibility and controlled trials.

## 9 Discussion

### 9.1 Key observations and interpretation

This narrative review integrates conceptual and theoretical perspectives, measurement approaches, and findings on flourishing in people living with chronic diseases. Three principal observations emerge from this synthesis. First, flourishing represents a multidimensional state of positive emotional, psychological, and social functioning rather than merely the absence of psychological distress. This distinction is particularly relevant in chronic disease care because psychological distress and positive functioning may coexist during adaptation to long-term illness. Second, Keyes’ framework and the PERMA model provide complementary theoretical foundations for understanding flourishing in chronic disease populations. Keyes’ framework distinguishes psychological distress from positive mental health, whereas PERMA specifies the domains of positive functioning, including positive emotion, engagement, relationships, meaning, and accomplishment. Together, these perspectives support a dual focus in chronic disease care: reducing psychological distress while also promoting positive functioning and flourishing. Third, the reviewed evidence suggests that flourishing is associated with both contextual and disease-related conditions and individual psychological resources. This synthesis underpins the proposed conceptual framework, which links contextual and disease-related factors, psychological resources, and two complementary pathways: distress reduction and the promotion of positive functioning. The framework therefore reflects the view that flourishing in chronic disease is associated not only with disease burden but also with patients’ social circumstances and available psychological resources.

### 9.2 Implications for research and practice

For research, greater conceptual precision is needed when selecting measures and comparing findings across studies. Researchers should state whether flourishing is being operationalized as positive mental health, PERMA-based functioning, global psychosocial flourishing, or broader human flourishing. Multiwave longitudinal studies are needed to characterize trajectories across diagnosis, treatment, recurrence, rehabilitation, and long-term survivorship. Studies should also examine whether psychological resources mediate the associations of social and disease-related factors with flourishing, and whether these pathways vary by disease type, age, socioeconomic circumstances, and cultural context. Beyond implications for research, VanderWeele and colleagues have advanced the integration of flourishing into medicine and public health by highlighting flourishing as an important goal of health beyond disease control and symptom reduction (VanderWeele et al., 2019; VanderWeele, 2024). Consistent with this perspective, our proposed framework extends this view to chronic disease populations by integrating contextual and disease-related conditions with psychological resources and linking them to two complementary pathways: distress reduction and promotion of positive functioning. For practice, the review supports a broader assessment perspective that considers psychological distress and positive functioning as related but distinct dimensions. However, the proposed framework should not yet be used as a prescriptive clinical pathway. Clinicians may use its domains to identify areas for discussion—such as meaning, relationships, engagement, coping, and accomplishment—while continuing to apply validated screening and treatment approaches for anxiety, depression, and other mental health problems.

### 9.3 Limitations

Several limitations should be acknowledged. First, this is a narrative rather than a systematic review; literature identification and selection may therefore be subject to omission and interpretive bias. Second, no formal risk-of-bias or certainty assessment was conducted, and evidence from studies with different designs and measurement approaches was synthesized qualitatively. Third, much of the available evidence is cross-sectional, which limits causal inference and the identification of temporal and mediating pathways. Fourth, evidence is unevenly distributed across disease populations, and a substantial proportion of the disease-specific evidence derives from single cross-sectional studies conducted in China, often in selected clinical samples. This concentration may limit the generalizability of the findings to other cultural and healthcare contexts. Fifth, the proposed framework has not undergone stakeholder consultation, formal consensus development, or empirical validation and should therefore be considered hypothesis-generating rather than a validated causal model or clinical pathway.

## 10 Conclusion

Flourishing provides a useful perspective for understanding how people may sustain positive emotional, psychological, and social functioning while living with chronic disease. The evidence base remains limited and uneven across disease populations, and findings are complicated by conceptual and measurement heterogeneity. Available studies nevertheless suggest that flourishing is associated with disease burden, social resources, psychological distress, and adaptive psychological resources.

Future research should use clearly specified constructs and validated instruments, recruit larger and more representative samples, and employ multiwave longitudinal and intervention designs. The proposed framework integrates Keyes’ model, PERMA-oriented domains, contextual factors, and psychological resources, but its directional relationships remain hypothetical. Stakeholder-informed conceptual validation and prospective empirical testing are required before it can guide a formal intervention program. Such work may ultimately support more person-centered approaches that address both psychological distress and positive functioning in chronic disease care.
